# Incidence, clinical features, and survival outcomes of primary malignant lacrimal gland tumors: A population‐based analysis

**DOI:** 10.1002/cam4.6831

**Published:** 2024-01-17

**Authors:** Lin‐feng He, Jin‐di Zhang, Teng‐fei Zhu, Peng‐cheng Zhao, Pei Mou, Shi‐yi Tang

**Affiliations:** ^1^ Department of Ophthalmology Changzheng Hospital, Second Affiliated Hospital of Naval Medical University Shanghai China; ^2^ Department of Anesthesiology Changzheng Hospital, Second Affiliated Hospital of Naval Medical University Shanghai China; ^3^ Department of Anesthesiology Shanghai Ninth People's Hospital, Shanghai Jiao Tong University School of Medicine Shanghai China; ^4^ Department of Ophthalmology Gongli Hospital of Shanghai Pudong New Area Shanghai China

**Keywords:** incidence, lacrimal gland tumors, prognosis, SEER

## Abstract

**Background:**

Studies on the epidemiological information and prognosis of primary malignant lacrimal gland tumors (MLGTs) are rare for its low occurrence. The goal of our research was to investigate the epidemiological characteristics and survival outcomes of patients with MLGTs.

**Methods:**

Incidence and demographic information of patients with MLGTs were collected from the Surveillance, Epidemiology, and End Results (SEER) database. To identify independent prognostic factors for disease‐specific survival (DSS) and overall survival (OS), univariate and multivariate Cox regression analysis were performed.

**Results:**

The overall incidence of primary MLGTs from 1975 to 2020 was 0.413/1,000,000 (according to the 2000 American standard population), with a steadily increasing incidence over years. A total of 964 patients with primary MLGTs were diagnosed, with an average age of 59.3 years. Of these, 53.2% were aged ≥60 years, 57.4% were female, and 77.1% were whites. Multivariate Cox regression analysis demonstrated that year of diagnosis, age, sex, histological type, SEER stage, surgery, and chemotherapy were independent prognostic factors of DSS or OS.

**Conclusions:**

Although primary MLGT is rare, its incidence has steadily increased in the past 46 years, and surgery was related to a better prognosis.

## 1 INTRODUCTION

Tumors of the lacrimal gland are rare, with an incidence of 1 case per million people per year in Denmark, and the incidence rate doubled during the study period.^1^ These lesions represent approximately 10% of orbital space‐occupying lesions, and the majority of them are benign.^2^, ^3^, ^4^ The most common benign tumor is pleomorphic adenoma, which accounts for nearly 50% of epithelial tumors.^5^ Other benign tumors, such as Warthin's tumor, oncocytoma, and myoepithelioma, are exceptionally scarce.^6^ Primary malignant lacrimal gland tumors (MLGTs) are fairly rare and half of them are epithelial tumors, while adenoid cystic carcinoma (ACC) is the most frequent type, followed by carcinoma ex pleomorphic adenoma, adenocarcinoma (ADC), and mucoepidermoid carcinoma (MEC).^1^, ^3^, ^7^, ^8^ Lymphoma (LYM), as the most common non‐epithelial tumors, account for 14% of lacrimal gland tumors.^3^ Other malignant tumor types, including squamous cell carcinoma (SCC), sebaceous carcinoma, and basal cell adenocarcinoma, have also been reported.^9^, ^10^, ^11^, ^12^, ^13^

The typical clinical symptoms of lacrimal gland tumors usually present with facial asymmetry due to the displacement of the eyeball, ptosis, decreased motility, and swelling of the lacrimal gland.^5^, ^14^ Pain is unusual in patients with benign tumors, but occurs more often in patients with MLGTs, especially in patients with ACC due to its characteristic perineural growth pattern.^5^, ^9^

The prognosis of MLGTs is poor, and the treatment still remains controversial, especially for ACC, and treatment regimens in advanced stages are often extrapolated from tumors in other sites.^8^, ^15^ Due to the rarity of primary MLGTs and the variety of histological types, the epidemiological information and survival outcomes of these patients are mainly based on case reports and small‐sample retrospective studies. Thus, this retrospective study aimed to describe the epidemiological characteristics and analyze the prognosis of primary MLGTs based on data from the Surveillance, Epidemiology, and End Results (SEER) database, which is an important resource of high‐quality data for cancer in the United States and covers nearly 35% of the US population.^16^

## 2 METHODS

### 2.1 Data source and cohort selection

A retrospective cohort analysis consisting of 964 patients diagnosed with MLGT was performed using SEER*Stat software (version 8.4.1.2). To enroll as many patients as possible, we collected patients between 1975 and 2019 through the SEER 17, 13, and 9 Registries.^17^, ^18^, ^19^ Inclusion criteria: (1) site‐specific code C69.6 (lacrimal gland) was used to extract patients with lacrimal gland malignancy; (2) diagnosis with microscopic confirmation; and (3) active follow‐up. Exclusion criteria: (1) survival month = 0; (2) diagnosis from autopsy or death certificate; (3) lacrimal duct and lacrimal sac according to the SEER coding scheme; (4) lacrimal gland tumor was not the first primary tumor. The detailed information extracted from SEER included age, year of diagnosis, sex (male and female), race (white, black and others), marital status (married and others), histological type, SEER stage (localized, regional, distant, and unknown), surgery (performed, no/unknown), radiotherapy (performed, no/unknown), and chemotherapy (performed, no/unknown). Year of diagnosis was grouped at 11‐year interval except for group from 1975 to 1986. On the basis of the third edition of *International Classification of Diseases for Oncology*, histological type was divided into six groups: ACC (8200/3), ADC (8140/3, 8290/3, 8410/3, 8481/3), SCC (8052/3, 8070/3, 8071/3, 8072/3, 8083/3), MEC (8430/3), LYM (959–972), and others. Annual incidence rates of primary MLGTs from 1975 to 2020 were obtained from SEER 8 Registries, and the rates were adjusted to the 2000 American standard population.^20^ Since the SEER program is publicly available and we obtained the access after completing its application process, an additional research ethics review was not needed.

### 2.2 Statistical analyses

The age‐adjusted incidence rates of primary MLGTs were displayed per 1,000,000 persons via SEER*Stat software (version 8.4.1.2). The annual percentage change (APC) and 95% confidence interval (CI) were obtained as well. All clinicopathological variables were changed into categorical variables and were displayed as frequencies. Statistical comparisons of variables were performed using chi‐square test or Fisher's exact test. Disease‐specific survival (DSS) was defined as the interval between the first date of diagnosis and death from primary disease. Overall survival (OS) was measured as the interval between the first date of diagnosis and death from any cause. DSS and OS were analyzed by Kaplan–Meier method, and differences between survival curves were calculated by the log‐rank test. Univariate and multivariate Cox proportional hazards models were performed to calculate hazard ratios and 95% confidence intervals for OS and DSS. Significant factors (*p* < 0.05) obtained in univariate Cox regression analysis and treatment modalities, which we were interested in, were incorporated into multivariate Cox regression analysis. Statistical analyses were employed using SPSS 26.0 (IBM) and R software (version 4.3.0).

## 3 RESULTS

### 3.1 Incidence of malignant lacrimal gland tumors

**FIGURE 1:**
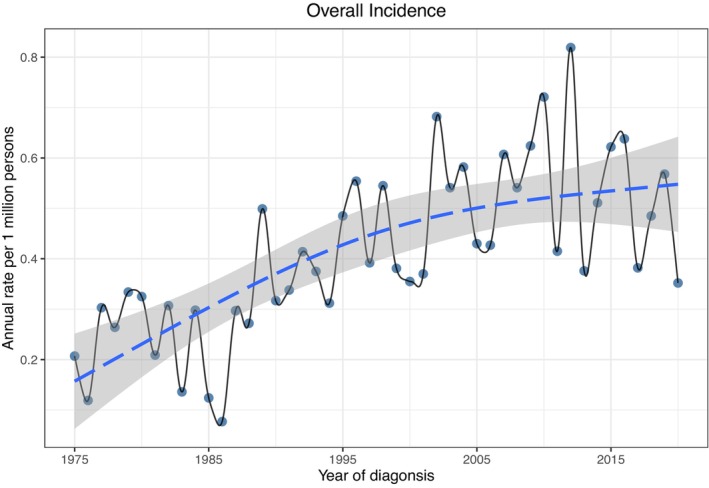
Incidence of malignant lacrimal gland tumors from 1975 to 2020 adjusted to the 2000 standard US population.

The overall incidence of primary MLGTs from 1975 to 2020 was 0.413 per 1,000,000 persons (APC 1.516, 95% CI 0.802–2.235, *p* < 0.05), with a steadily increasing incidence over the years (Figure 1). The incidence in people aged ≥60 years (1.398) was prominently higher than that in those aged <60 years (0.218). For the sex group, the incidence of MLGTs in females (0.436) was higher than that in males (0.384). Analysis by race showed that the highest incidence rate was observed in blacks (0.503), and whites had the lowest incidence (0.396) with an APC of 1.522 (95% CI 0.671–2.381, *p* < 0.05). Among the common histological subtypes of primary MLGTs, LYM had the highest incidence rate (0.221), followed by ACC (0.063) (Table 1).

**TABLE 1:** Incidence rate from 1975 to 2020.

	Incidence rate	APC
Overall	0.413	1.516^a^ (0.802–2.235)
Age		
<60	0.218	NA
≥60	1.398	NA
Sex		
Male	0.384	NA
Female	0.436	NA
Race		
White	0.396	1.522^a^ (0.671–2.381)
Black	0.503	NA
Others^b^	0.455	NA
Histological type		
ACC	0.063	NA
ADC	0.017	NA
SCC	0.045	NA
MEC	0.016	NA
LYM	0.221	NA

^a^ The APC is significantly different from zero (*p* < 0.05).

^b^ American Indian/AK Native, Asian/Pacific Islander, and unknown.

### 3.2 Demographics of patients with malignant lacrimal gland tumor

Following strict inclusion and exclusion criteria, a total of 964 patients with MLGTs, diagnosed between 1975 and 2019, were included in this study. The mean age of the 964 patients was 59.3 ± 16.9 years old (range, 6–101 years), and 513 (53.2%) patients were aged ≥60 years. The mean age of patients with ACC was much younger (49.1 years) than the overall mean age, while the mean age of patients with LYM was much older (62.1 years). Of these patients, 411 (42.6%) were males, 553 (57.4%) were females, and the majority were White (77.1%). The most common MLGT was LYM (56.8%), followed by ACC (13.5%), SCC (11.2%), ADC (4%), MEC (3.1%), and other histological types (11.3%). Regarding the SEER summary stage, the proportion of localized stage was 24.3%, and the proportions of regional, distant, and unknown stage were 16.6%, 8.3%, and 50.8% respectively. Concerning treatment modalities, patients with MLGTs were more likely to receive radiotherapy (55%) and surgery (48.4%), but less likely to receive chemotherapy (21.2%). Among the different histological subtypes, the proportion of surgery in patients with LYM (33%) was much less than that in the others (Table 2).

**TABLE 2:** Demographic and clinical characteristics of patients.

Variables	Total	ACC	ADC	SCC	MEC	LYM	Other	*p*
Number of patients (%)	964	130 (13.5%)	39 (4%)	108 (11.2%)	30 (3.1%)	548 (56.8%)	109 (11.3%)	
Age								
Mean (SD)	59.3 (16.9)	49.1 (18.5)	61.1 (14.2)	59.5 (12.5)	54.5 (16.1)	62.1 (15.8)	58.1 (19.5)	<0.001
Median (first quartile, third quartile)	61[49,71]	52[35,64]	64[49,71]	59[50.25,68]	58.5[43.25,65.25]	63[51.25,73]	60[45,72]	
<60	451 (46.8%)	89 (68.5%)	11 (28.2%)	57 (52.8%)	16 (53.3%)	228 (41.6%)	50 (45.9%)	
≥60	513 (53.2%)	41 (31.5%)	28 (71.8%)	51 (47.2%)	14 (46.7%)	320 (58.4%)	59 (54.1%)	
Year of diagnosis								
1975–1986	56 (5.8%)	20 (15.4%)	7 (17.9%)	5 (4.6%)	2 (6.7%)	16 (2.9%)	6 (5.5%)	<0.001
1987–1997	121 (12.6%)	14 (10.8%)	5 (12.8%)	16 (14.8%)	4 (13.3%)	74 (13.5%)	8 (7.3%)	
1998–2008	367 (38.1%)	39 (30%)	10 (25.6%)	46 (42.6%)	13 (43.3%)	218 (39.8%)	41 (37.6%)	
2009–2019	420 (43.6%)	57 (43.8%)	17 (43.6%)	41 (38%)	11 (36.7%)	240 (43.8%)	54 (49.5%)	
Sex								
Male	411 (42.6%)	55 (42.3%)	30 (76.9%)	67 (62%)	16 (53.3%)	182 (33.2%)	61 (56%)	<0.001
Female	553 (57.4%)	75 (57.7%)	9 (23.1%)	41 (38%)	14 (46.7%)	366 (66.8%)	48 (44%)	
Race								
White	743 (77.1%)	86 (66.2%)	31 (79.5%)	85 (78.7%)	17 (56.7%)	441 (80.5%)	83 (76.1%)	<0.001
Black	96 (10%)	25 (19.2%)	1 (2.6%)	10 (9.3%)	4 (13.3%)	39 (7.1%)	17 (15.6%)	
Others^a^	125 (13%)	19 (14.6%)	7 (17.9%)	13 (12%)	9 (30%)	68 (12.4%)	9 (8.3%)	
Marital status								
Married	525 (54.5%)	66 (50.8%)	23 (59%)	60 (55.6%)	14 (46.7%)	307 (56%)	55 (50.5%)	0.69
Other	439 (45.5%)	64 (49.2%)	16 (41%)	48 (44.4%)	16 (53.3%)	241 (44%)	54 (49.5%)	
SEER stage								
Localized	234 (24.3%)	50 (38.5%)	14 (35.9%)	50 (46.3%)	15 (50%)	65 (11.9%)	40 (36.7%)	<0.001
Regional	160 (16.6%)	54 (41.5%)	20 (51.3%)	42 (38.9%)	10 (33.3%)	5 (0.9%)	29 (26.6%)	
Distant	80 (8.3%)	18 (13.8%)	5 (12.8%)	11 (10.2%)	4 (13.3%)	21 (3.8%)	21 (19.3%)	
Unknown	490 (50.8%)	8 (6.2%)	0	5 (4.6%)	1 (3.3%)	457 (83.4%)	19 (17.4%)	
Surgery								
Performed	467 (48.4%)	87 (66.9%)	25 (64.1%)	75 (69.4%)	23 (76.7%)	181 (33%)	76 (69.7%)	<0.001
No/unknown	497 (51.6%)	43 (33.1%)	14 (35.9%)	33 (30.6%)	7 (23.3%)	367 (67%)	33 (30.3%)	
Radiotherapy								
Performed	530 (55%)	81 (62.3%)	21 (53.8%)	64 (59.3%)	16 (53.3%)	292 (53.3%)	56 (51.4%)	0.431
No/unknown	434 (45%)	49 (37.7%)	18 (46.2%)	44 (40.7%)	14 (46.7%)	256 (46.7%)	53 (48.6%)	
Chemotherapy								
Performed	204 (21.2%)	27 (20.8%)	10 (25.6%)	12 (11.1%)	2 (6.7%)	137 (25%)	16 (14.7%)	0.003
No/unknown	760 (78.8%)	103 (79.2%)	29 (74.4%)	96 (88.9%)	28 (93.3%)	411 (75%)	93 (85.3%)	

^a^ American Indian/AK Native, Asian/Pacific Islander, and unknown.

### 3.3 Survival analysis

**FIGURE 2:**
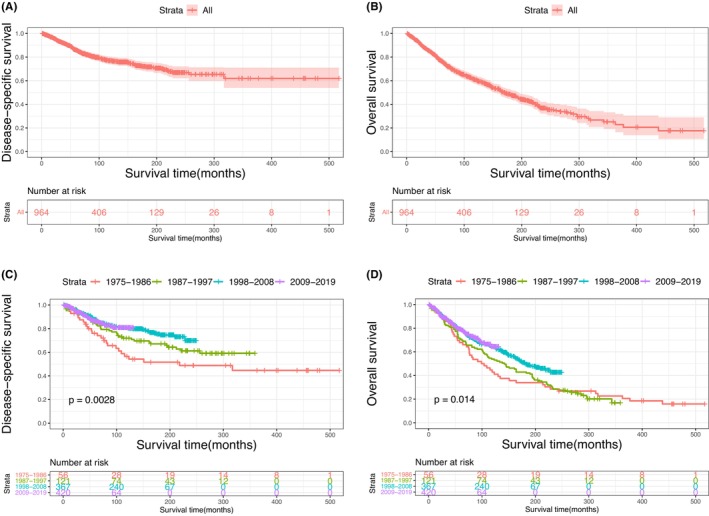
Disease‐specific survival (A) and overall survival (B) of malignant lacrimal gland tumors; disease‐specific survival (C) and overall survival (D) according to years of diagnosis.

**FIGURE 3:**
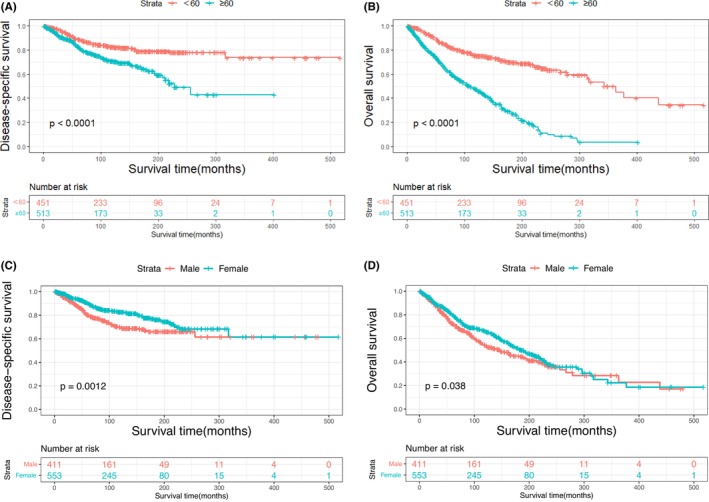
Disease‐specific survival according to age (A), and sex (C); overall survival according to age (B), and sex (D).

**FIGURE 4:**
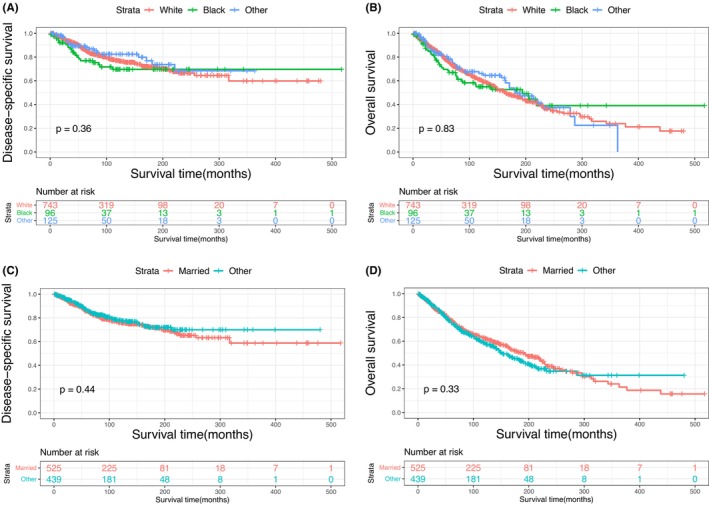
Disease‐specific survival according to race (A), and marital status (C); overall survival according to race (B), and marital status (D).

**FIGURE 5:**
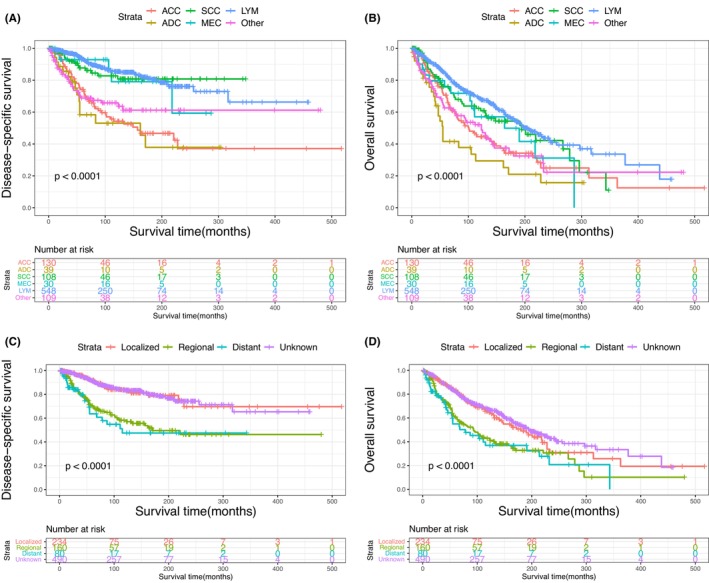
Disease‐specific survival according to histological type (A), and SEER stage (C); overall survival according to histological type (B), and SEER stage (D).

**FIGURE 6:**
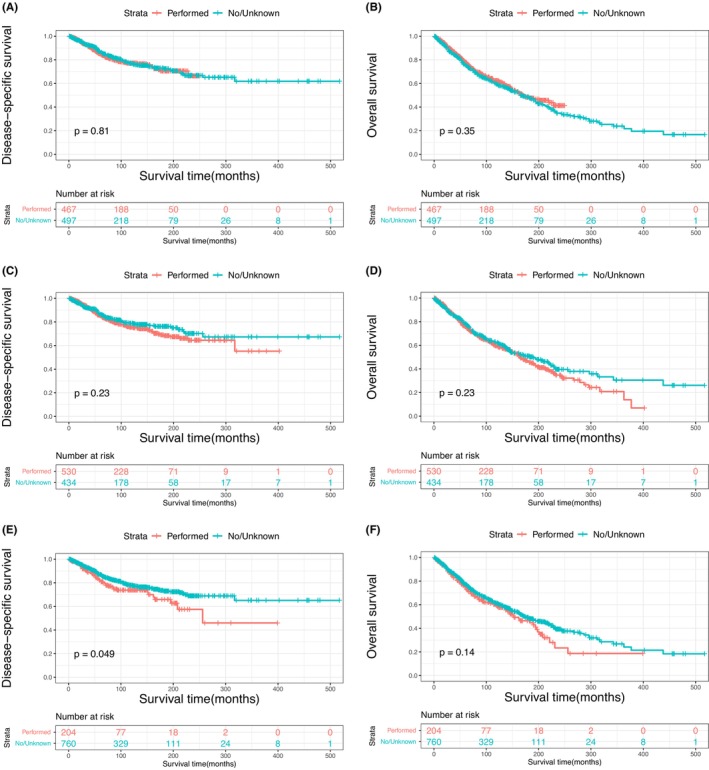
Disease‐specific survival according to surgery (A), radiotherapy (C), and chemotherapy (E); overall survival according to surgery (B), radiotherapy (D), and chemotherapy (F).

The Kaplan–Meier plots of DSS and OS for patients with primary MLGTs and years of diagnosis are shown in Figure 2. A total of 401 patients died at the end of the study, among them 188 died of MLGTs. The prognosis has improved over the years. The DSS and OS of patients identified in 2009–2019, 1998–2008, and 1987–1997 were significantly improved compared to those of patients identified in 1975–1986. The survival curves for age, sex, race, marital status, histological type, SEER stage, and treatment methods were also plotted based on the Kaplan–Meier analysis. Patients aged ≥60 and male patients had lower DSS and OS (Figure 3). There was no significant difference in DSS and OS between the race and the marital status groups (Figure 4). In terms of tumor characteristics, patients with lacrimal gland LYM and localized tumors showed prolonged survival (Figure 5). Regarding treatment methods, including surgery, radiotherapy, and chemotherapy, none of them were associated with better prognoses based on Kaplan–Meier analysis (Figure 6).

Cox regression analyses were performed to identify independent prognostic factors for DSS and OS. The analysis demonstrated that year of diagnosis, age, sex, histological type, SEER stage, and chemotherapy independently predicted DSS (Table 3). Meanwhile, age, histological type, SEER stage, surgery, and chemotherapy were significantly associated with OS (Table 4). Subgroup analyses according to histological types were performed, which demonstrated that surgery was related to better DSS and OS in lacrimal gland LYM and related to better DSS in SCC, as well as better OS in ACC (Data S1).

**TABLE 3:** The results of the univariate and multivariate Cox regression analysis for disease‐specific survival.

Variables	Univariate analysis		Multivariate analysis	
	HR (95% CI)	*p* Value	HR (95% CI)	*p* Value
Year of diagnosis				
1975–1986	Ref	0.04	Ref	0.039
1987–1997	0.654 (0.397–1.08)	0.097	0.726 (0.426–1.237)	0.239
1998–2008	0.453 (0.289–0.71)	0.001	0.57 (0.351–0.925)	0.023
2009–2019	0.481 (0.293–0.79)	0.004	0.481 (0.286–0.811)	0.006
Age				
<60	Ref	<0.001	Ref	<0.001
≥60	2.043 (1.514–2.757)		2.611 (1.903–3.582)	
Sex				
Male	Ref	0.001	Ref	0.031
Female	0.626 (0.47–0.834)		0.717 (0.529–0.971)	
Race				
White	Ref	0.366		
Black	1.282 (0.819–2.007)			
Others^a^	0.833 (0.522–1.329)			
Marital status				
Married	Ref	0.441		
Other	0.892 (0.667–1.193)			
Histological type				
ACC	Ref	<0.001	Ref	<0.001
ADC	1.198 (0.673–2.132)	0.539	0.748 (0.411–1.362)	0.342
SCC	0.315 (0.174–0.569)	<0.001	0.274 (0.15–0.5)	<0.001
MEC	0.361 (0.144–0.904)	0.03	0.331 (0.131–0.837)	0.02
LYM	0.279 (0.194–0.401)	<0.001	0.141 (0.071–0.278)	<0.001
Others	0.838 (0.543–1.293)	0.425	0.635 (0.4–1.009)	0.055
SEER stage				
Localized	Ref	<0.001	Ref	<0.001
Regional	3.074 (1.925–4.908)	<0.001	2.611 (1.621–4.207)	<0.001
Distant	3.592 (2.051–6.292)	<0.001	3.544 (2.006–6.262)	<0.001
Unknown	1.026 (0.654–1.608)	0.912	3.199 (1.601–6.393)	0.001
Surgery				
Performed	Ref	0.814		0.836
No/unknown	0.966 (0.724–1.289)			
Radiotherapy				
Performed	Ref	0.235		0.124
No/unknown	0.838 (0.626–1.122)			
Chemotherapy				
Performed	Ref	0.05	Ref	0.003
No/unknown	0.72 (0.518–1)		0.587 (0.413–0.834)	

^a^ American Indian/AK Native, Asian/Pacific Islander, and unknown.

**TABLE 4:** The results of the univariate and multivariate Cox regression analysis for overall survival.

Variables	Univariate analysis		Multivariate analysis	
	HR (95% CI)	*p* Value	HR (95% CI)	*p* Value
Year of diagnosis				
1975–1986	Ref	0.015		0.549
1987–1997	0.983 (0.683–1.415)	0.927		
1998–2008	0.696 (0.494–0.981)	0.039		
2009–2019	0.675 (0.458–0.992)	0.046		
Age				
<60	Ref	<0.001	Ref	<0.001
≥60	3.521 (2.815–4.404)		3.899 (3.101–4.904)	
Sex				
Male	Ref	0.039		0.125
Female	0.812 (0.667–0.989)			
Race				
White	Ref	0.834		
Black	1.048 (0.752–1.463)	0.78		
Others^a^	0.926 (0.683–1.255)	0.62		
Marital status				
Married	Ref	0.327		
Other	1.104 (0.906–1.344)			
Histological type				
ACC	Ref	<0.001	Ref	<0.001
ADC	1.51 (0.962–2.37)	0.074	0.976 (0.617–1.542)	0.916
SCC	0.703 (0.482–1.026)	0.068	0.557 (0.38–0.815)	0.003
MEC	0.791 (0.452–1.382)	0.409	0.591 (0.336–1.04)	0.068
LYM	0.552 (0.42–0.727)	<0.001	0.296 (0.177–0.496)	<0.001
Others	1.02 (0.719–1.447)	0.911	0.731 (0.508–1.052)	0.091
SEER stage				
Localized	Ref	<0.001	Ref	0.001
Regional	1.745 (1.281–2.378)	<0.001	1.545 (1.13–2.111)	0.006
Distant	1.989 (1.336–2.962)	0.001	2.219 (1.483–3.322)	<0.001
Unknown	0.903 (0.688–1.184)	0.46	1.604 (0.974–2.641)	0.063
Surgery				
Performed	Ref	0.354	Ref	0.01
No/unknown	1.1 (0.899–1.347)		1.335 (1.073–1.66)	
Radiotherapy				
Performed	Ref	0.233		0.088
No/unknown	0.886 (0.726–1.081)			
Chemotherapy				
Performed	Ref	0.138	Ref	0.044
No/unknown	0.836 (0.659–1.06)		0.776 (0.607–0.993)	

^a^ American Indian/AK Native, Asian/Pacific Islander, and unknown.

## 4 DISCUSSION

Owing to its rarity, there are very few studies on the incidence, clinicopathological characteristics, and survival outcomes of MLGTs. Thus, the current study conducted a population‐based cohort study to analyze the epidemiology and prognosis of patients with primary MLGTs by using SEER database. We calculated the incidence trends and overall incidence of primary MLGTs and assessed their clinicopathological parameters, treatment modalities, and independent prognostic factors. Based on the study, several important conclusions can be derived.

Our study showed that the overall incidence of primary MLGTs from 1975 to 2020 was 0.413 per million individuals, which was slightly higher than that in Denmark, and the trend in incidence rate was relatively steadily increasing in the last four decades.^21^ Specific reasons for the increasing trend in incidence cannot be derived from this study but may be partly attributed to better imaging modalities such as high‐resolution computerized tomography and magnetic resonance imaging, as well as the improved knowledge of this disease.^22^ In addition, the trend in the incidence of ophthalmic LYM seems to be continually increasing.^23^ The median age at diagnosis of primary MLGTs in this cohort was 61 years, and the incidence rate of the elderly was nearly seven times more than that in people aged under 60 years old, which is line with previous studies.^1^, ^24^ Consistent with other studies, the mean age of patients with LYM was older than that of patients with other subtypes, while patients with ACC was much younger.^5^, ^7^, ^25^, ^26^ Advanced age was related to worse DSS and OS. Elderly patients usually have more comorbidities compared with young patients, which may impact their survival outcomes directly and treatment modalities for low tolerability.^27^ The incidence rate of MLGTs in females was higher than that in males. In a study of lacrimal gland LYM, Rasmussen and his colleagues detected that females had a predominance compared with males; however, a multicenter retrospective study demonstrated an opposite predominance.^28^, ^29^ The survival analysis showed that females were related to better DSS, which indicated that males with MLGTs need more attention.

LYM was the most common histological subtype in the present study, which is consistent with the study reported by Sjo^30^ that LYM was the most frequent malignant tumor of ocular adnexa. ACC, the most common malignant epithelial tumor of the lacrimal gland, was the second most common histological subtype in our study, followed by SCC, ADC, and MEC.^5^ LYM had better DSS and OS, while ACC and ADC were substantially associated with worse prognoses, which is similar to previous studies.^29^, ^31^ A multicenter retrospective study conducted by Vest SD also showed that LYM of the lacrimal gland had a good prognosis for extranodal marginal zone B‐cell LYM accounting for approximately 70% of LYM subtypes.^29^ ACC carried an extremely poor prognosis for high rates of perineural and/or osseous invasion with intracranial extension and hematogenous or lymphatic spread.^32^, ^33^ Moreover, despite with the same pathological characteristics, ACC in eye and orbit seems to be related to worse survival compared with ACC in other common sites, such as salivary glands.^34^ ADC is also an aggressive tumor with a tendency to metastasize, and the mean survival time was only 3.5 years in 19 patients with ADC of lacrimal gland.^35^

There was an improvement in  prognosis over the years, which might benefit from the development of diagnostic methods and current comprehensive treatment modalities. In comparison with the patients diagnosed in 1975–1986, DSS and OS for patients diagnosed in 1987–1997, 1998–2008, and 2009–2019 were all greatly improved. Of note, the addition of anti‐CD20 monoclonal antibodies (such as rituximab) has improved complete remission of CD20‐positive B LYM since the 2000s.^36^, ^37^ Meanwhile, neoadjuvant intracarotid chemotherapy has achieved encouraging therapeutic effect since it was proposed for ACC.^38^, ^39^ The understanding of the molecular characteristics of MLGTs has improved, which has promoted new targeted drugs for patients with nonresectable or metastatic tumors. Multimodality managements, including surgery, radiotherapy, and adjuvant and neoadjuvant chemotherapy, also contribute to the progress. In terms of disease stage, the results showed that the SEER stage was associated with DSS and OS. Although TNM stage is widely used in clinical practice, SEER database does not document this information comprehensively. In addition, SEER stage is characterized as a standardized and simplified coding rules and provides consistent definitions over years.^40^ Consistent with previous studies, advanced stage was related to the high rate of recurrence and poor prognosis.^6^, ^29^, ^41^

The rarity of MLGTs combined with large spectrum of lesions makes the establishment of optimal management for every individual patient difficult. Due to the embryological and morphological similarities between the lacrimal gland and the salivary glands, treatment strategies of MLGTs are extrapolated from clinical studies of patients with salivary gland tumors.^21^, ^42^ Surgery, radiotherapy, and chemotherapy are performed independently or in combination in the treatment of MLGTs. In the present study, survival analysis showed that surgery was markedly associated with prolonged OS. A prior study conducted by Mallen‐St Clair et al.^43^ also revealed that surgical therapy acted as therapeutic determinant of survival in patients with lacrimal gland carcinoma. Surgical removal is the primary treatment for malignant epithelial tumor of the lacrimal gland, and the classical surgical approach is through lateral orbitotomy with removal of the lateral wall of the orbit.^21^ The tumor ought to, if the mass is circumscribed and small, be removed completely through lateral orbitotomy, which provides superb exposure; meanwhile, orbital exenteration should be taken into consideration when the mass is extensive and infiltrated beyond its capsule.^6^ Previous studies showed that ACC typically presented early microinvasion of neighboring tissues, which makes the mass difficult to evaluate during surgery, so orbital exenteration is indicated as a preferred treatment.^1^, ^22^ However, orbital exenteration has not been proven to be convincingly correlated with prolonged survival. Wright et al.^9^ reported that the disease‐free survival rate of ACC seemed to be irrelevant to cranio‐orbital resection. Hung et al.^44^ demonstrated that eye‐sparing surgery combined with adjunctive radiotherapy may control early‐stage ACC. Likewise, in a retrospective analysis on long‐term outcomes of patients with ACC, the authors detected that eye‐sparing surgery with adjuvant radiotherapy was an optional treatment for orbit‐confined ACC.^45^ Malignant epithelial tumors other than ACC are rare, and complete surgical resection, lower stage, and adjuvant treatments are associated with good prognoses.^22^ Complete tumor excision with or without radiotherapy may be viable for low‐grade and early‐stage ADC and MEC.^15^, ^46^ Consistent with previous studies, surgery is not usually chosen in the treatment of lacrimal gland LYM. It may be performed when the tumor size needs to be reduced, especially in the patient with a massive tumor volume load.^29^, ^47^ In the further subgroup  analysis according to histological type, surgery was associated with better DSS and OS in lacrimal gland LYM. Although surgery is not the cornerstone in the treatment of LYM, surgical excision is still appropriate for localized indolent LYM on account of the advantages of local disease control and local symptoms relief.^35^, ^48^, ^49^

In the present research, over half of the patients with MLGTs received radiotherapy. Radiation therapy can be performed to eradicate tumor cells, and it can also be used to reduce tumor size preoperatively. Postoperative radiation therapy and surgery are commonly used as initial local managements in patients with epithelial MLGTS, meanwhile radiotherapy is appropriate for patients with local and low‐grade LYM of lacrimal gland.^30^, ^50^

Besides the therapies discussed above, chemotherapy has played an important role in recent years for the treatment of MLGTs. Platinum‐based chemotherapy, which is commonly used in head and neck SCC, may be performed to improve the sensitivity of postoperative radiotherapy in patients with high recurrence risk malignant epithelial tumors.^51^ A long‐term follow‐up study showed that the implementation of neoadjuvant intra‐arterial cytoreductive chemotherapy seemed to improve OS and decrease recurrence in patients with lacriaml ACC.^52^ While Maniar and colleagues^53^ reported that a novel intra‐arterial chemotherapy technique utilizing both the external and internal carotid circulation, which may improve the prognosis of patients treated with eye‐sparing management.

Multidisciplinary teams are necessary for patients with MLGTs, since treatment for MLGTs varies by different institutions, and cancer management involves integrated multimodal strategies that require diverse medical specialties. Savino et al.^54^ designed an ocular oncology multidisciplinary tumor board (MDTB), involving ocular oncologists, ophthalmologists, medical oncologists, radiation oncologists, radiologists, maxillofacial surgeons, and otolaryngologists, pathologists, as well as a MDTB meeting coordinator and a clinical trial coordinator, which made positive contribution to comprehensive evaluation and integrated therapeutic management for oncologic patients. This board also streamlined the patient treatment process and might improve the prognosis of oncologic patients.

Several limitations ought to be noted in this research. First, as a retrospective study, inherent bias is inevitable. However, due to the rarity of this disease, large prospective clinical studies seem impossible. Although, we collected as many patients as possible from the SEER database, the number of patients was insufficient that some factors related to prognoses could not be analyzed well. Second, the period of patients with MLGTs enrolled in this study was long (45 years), treatment modalities and detection methods have changed. Third, detailed data on some variables, such as radiotherapy regimen, surgical approach, and chemotherapy regimen, are not acquirable. Therefore, it was difficult to accurately evaluate the influence on treatment, and the results should be interpreted carefully. Fourth, even though we excluded tumors of the lacrimal duct and lacrimal sac according to the SEER coding scheme, we could not ensure that all the tumors were derived from the lacrimal gland itself. Despite these limitations, this research provided important insights for primary MLGTs and useful information on the incidence, prognostic factors, and survival outcomes.

## 5 CONCLUSIONS

The present study showed that primary MLGTs are relatively rare tumors with an overall steadily increasing incidence during the past four decades. We also displayed the demographic characteristics of the main subtypes of MLGTs. Age at diagnosis, histological type, and SEER stage were identified as independent prognostic factors for both DSS and OS. Surgery was related to prolonged OS in patients with MLGTs, and surgical method might be considered positively in the patients with MLGTs. This research may provide novel insights into the treatment management and healthcare for patients with primary MLGTs.

## AUTHOR CONTRIBUTIONS

**Lin‐feng He:** Data curation (equal); methodology (equal); writing – original draft (equal). **Jin‐di Zhang:** Data curation (equal); methodology (equal). **Teng‐fei Zhu:** Data curation (equal); resources (equal). **Peng‐cheng Zhao:** Supervision (equal). **Pei Mou:** Supervision (equal). **Shi‐yi Tang:** Supervision (equal).

## FUNDING INFORMATION

This research received no funding.

## CONFLICT OF INTEREST STATEMENT

The authors declare that they have no competing interests.

## Supporting information


Data S1.

## Data Availability

All the data can be extracted from the SEER database (https://seer.cancer.gov/).
