# Supplementary material for: Incidence, clinical features, and survival outcomes of primary malignant lacrimal gland tumors: A population‐based analysis
Source: Cancer Med. 2024 Jan 17;13(3):e6831. doi: 10.1002/cam4.6831 (PMC10905223; doi:10.1002/cam4.6831)

Subgroup analyses according to histological types were performed to investigate the relationship between surgery and disease-specific survival.

| histological types | | chi-square | P |
| --- | --- | --- | --- |
| adenoid cystic carcinoma | Log Rank (Mantel-Cox) | 1.908 | .167 |
| adenocarcinoma | Log Rank (Mantel-Cox) | .310 | .578 |
| squamous cell carcinoma | Log Rank (Mantel-Cox) | 4.893 | .027 |
| mucoepidermoid carcinoma | Log Rank (Mantel-Cox) | .679 | .410 |
| lymphoma | Log Rank (Mantel-Cox) | 4.726 | .030 |
| others | Log Rank (Mantel-Cox) | 1.543 | .214 |


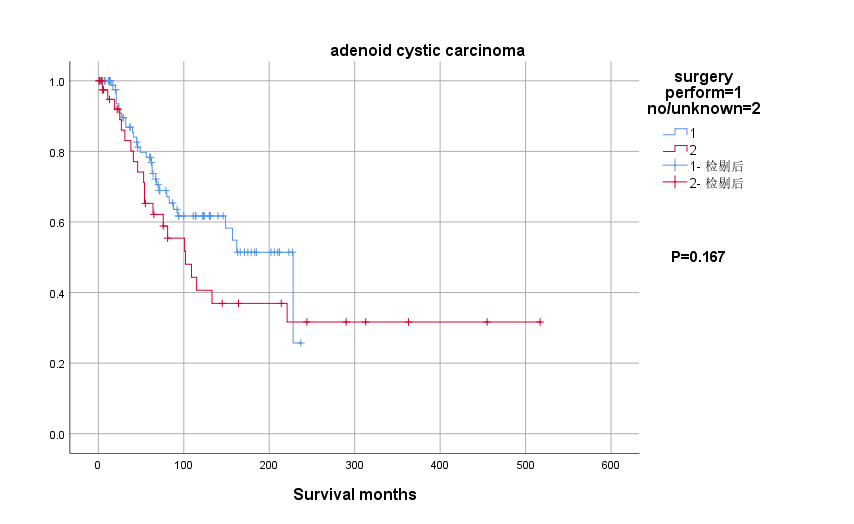


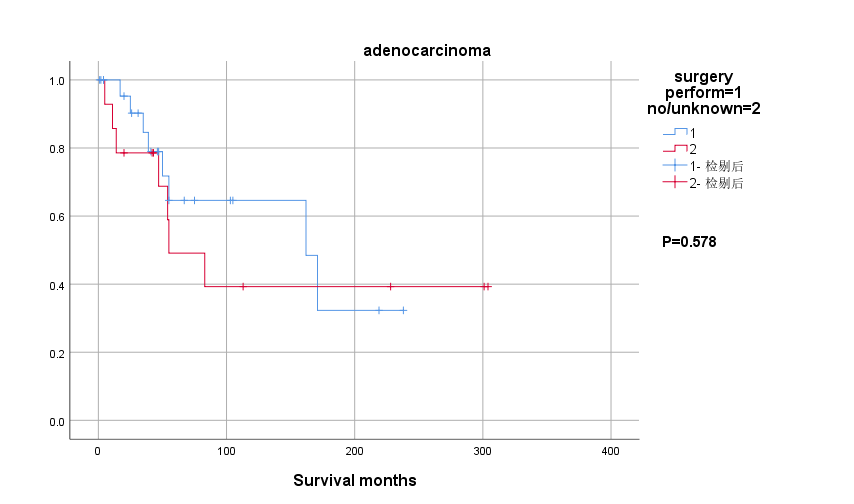


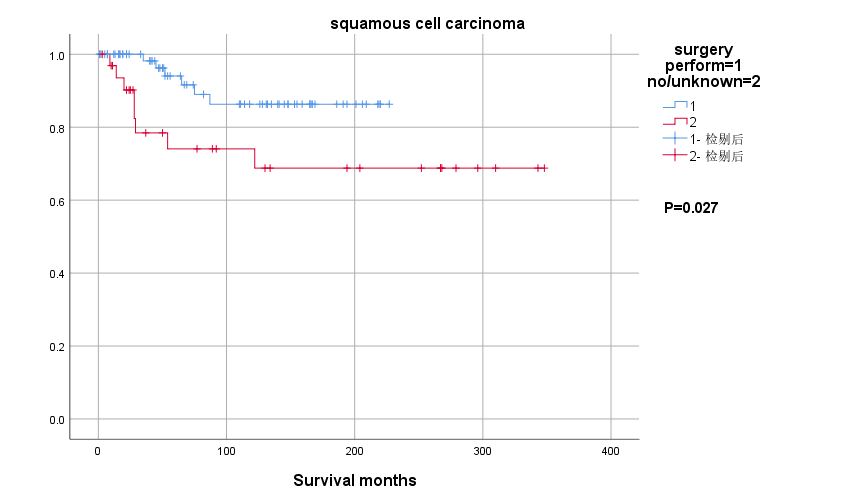

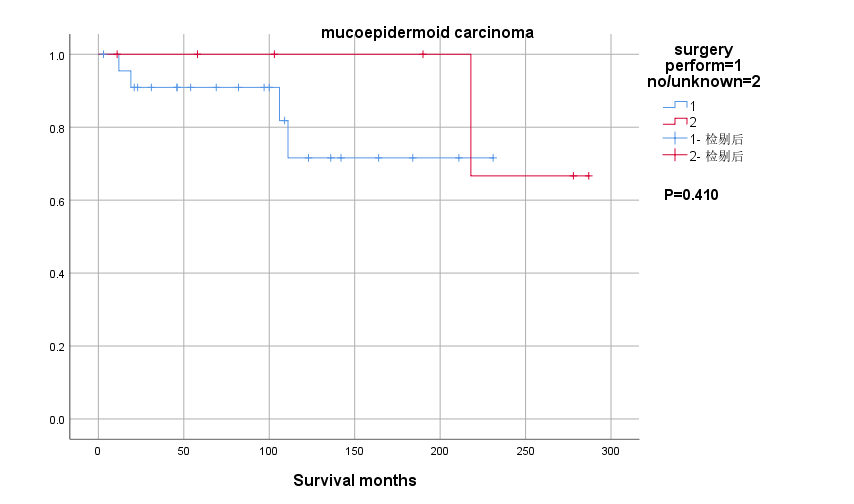

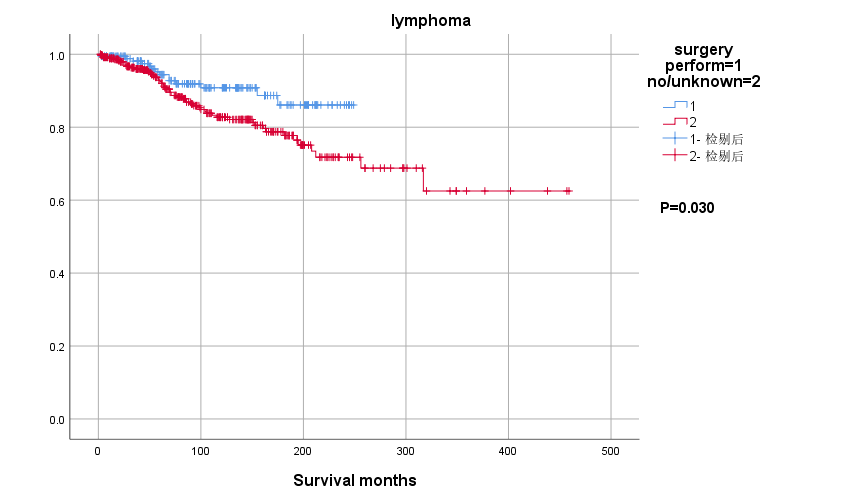

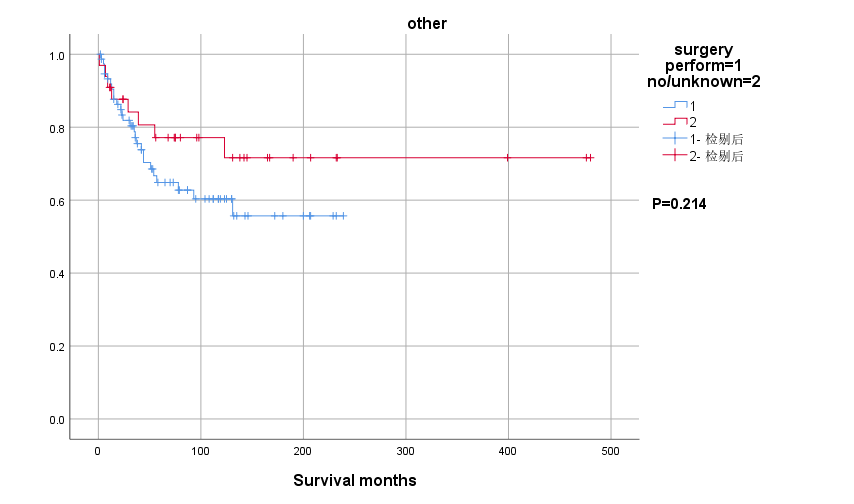


Subgroup analyses according to histological types were performed to investigate the relationship between surgery and overall survival.

| histological types | | chi-square | P |
| --- | --- | --- | --- |
| adenoid cystic carcinoma | Log Rank (Mantel-Cox) | 5.454 | .020 |
| adenocarcinoma | Log Rank (Mantel-Cox) | .012 | .914 |
| squamous cell carcinoma | Log Rank (Mantel-Cox) | 3.609 | .057 |
| mucoepidermoid carcinoma | Log Rank (Mantel-Cox) | .010 | .920 |
| lymphoma | Log Rank (Mantel-Cox) | 7.280 | .007 |
| others | Log Rank (Mantel-Cox) | 3.253 | .071 |


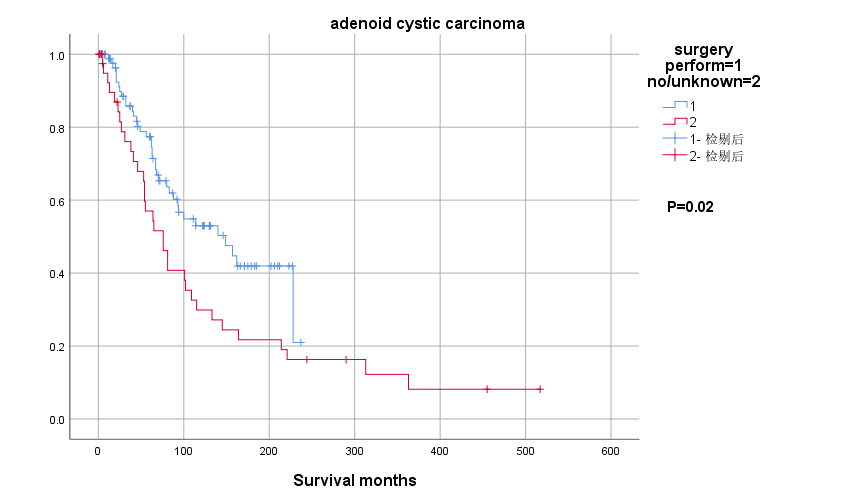


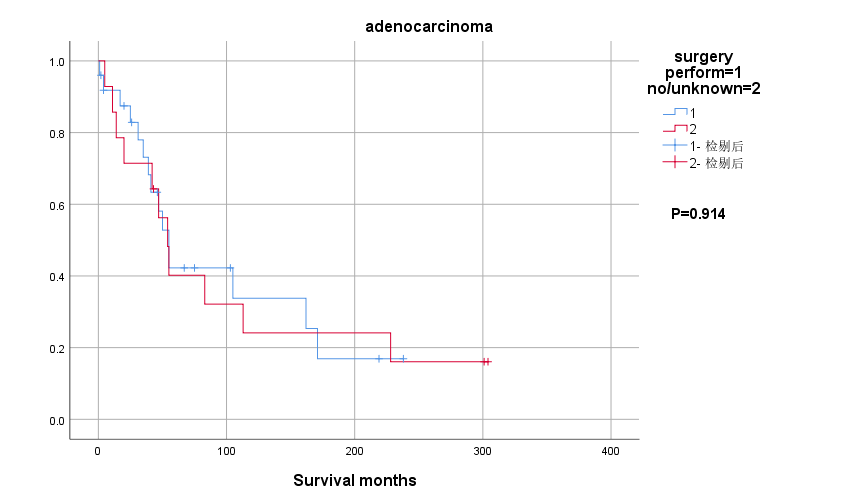

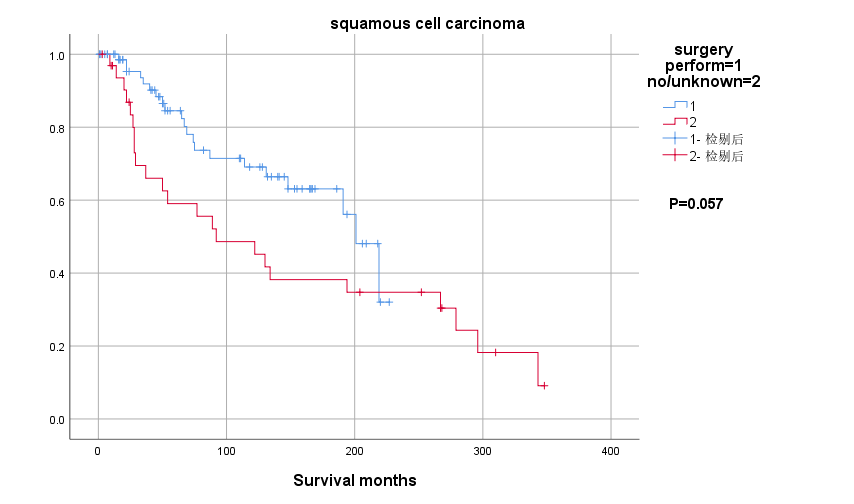

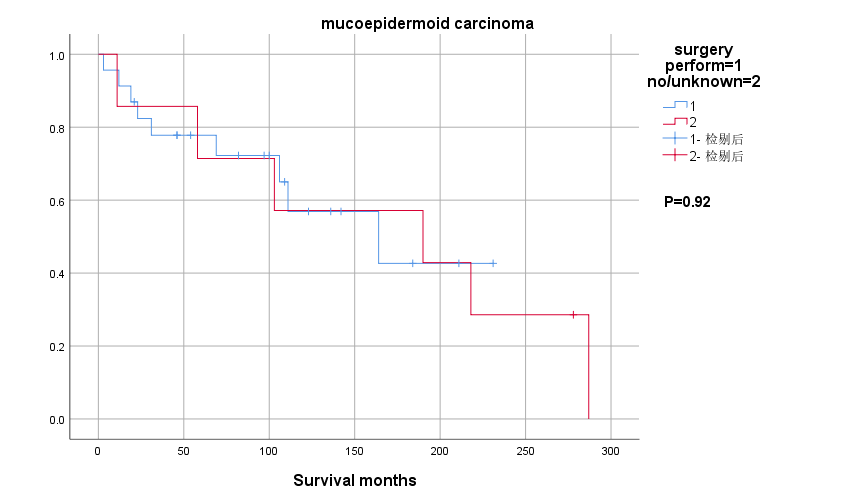

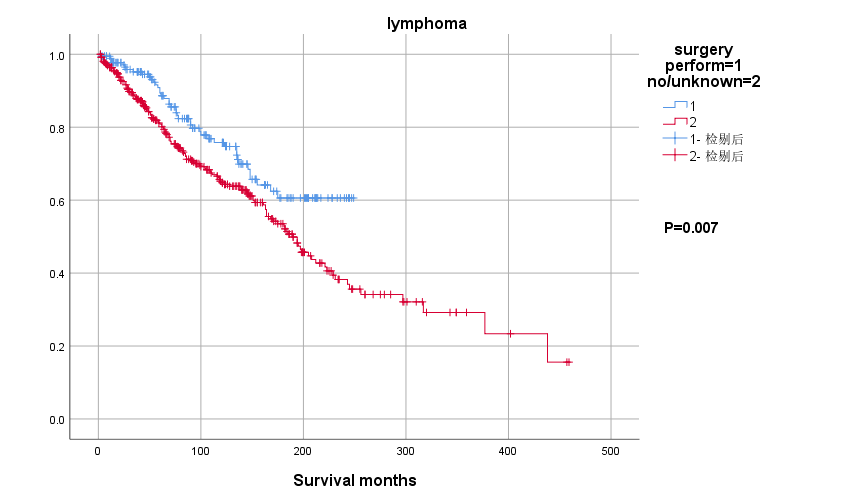

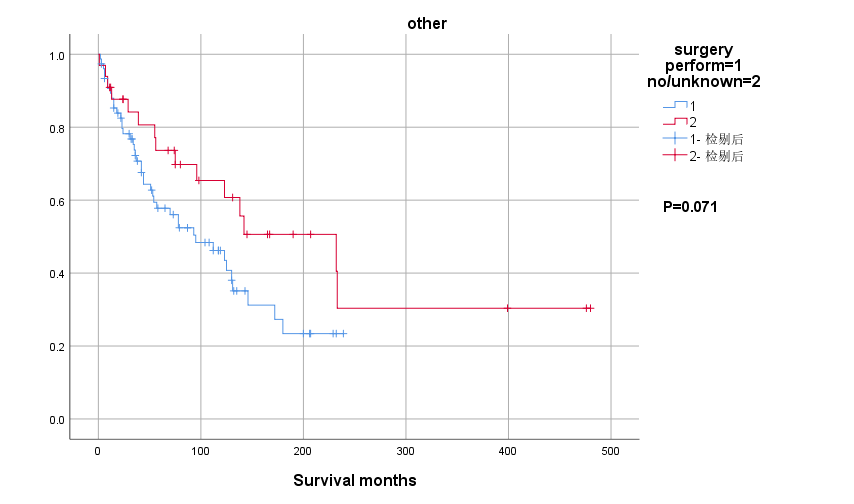

Supplement: Supplementary file 1 — Data S1. [file CAM4-13-e6831-s001.docx]
